# Biomass-Haze PM2.5 from Northern Thailand Drives Genotype-Specific Oxidative Stress and Transcriptomic Remodeling in Non-Small-Cell Lung Cancer Cells

**DOI:** 10.3390/toxics14010021

**Published:** 2025-12-25

**Authors:** Sakawwarin Prommana, Sitthisak Intarasit, Saruda Thongyim, Nuttipon Yabueng, Somporn Chantara, Pachara Sattayawat, Aussara Panya, Sahutchai Inwongwan

**Affiliations:** 1 Department of Biology, Faculty of Science, Chiang Mai University, Chiang Mai 50200, Thailand; sakawwarin_pr@cmu.ac.th (S.P.); sitthisak.inta@cmu.ac.th (S.I.); pachara.sattayawat@cmu.ac.th (P.S.); 2 Cell Engineering for Cancer Therapy Research Group, Chiang Mai University, Chiang Mai 50200, Thailand; supawadeethongyim@gmail.com; 3 Office of Research Administration, Chiang Mai University, Chiang Mai 50200, Thailand; 4 Environmental Science Research Center, Faculty of Science, Chiang Mai University, Chiang Mai 50200, Thailand; nuttipon.y@cmu.ac.th (N.Y.); somporn.chantara@cmu.ac.th (S.C.)

**Keywords:** biomass-haze PM2.5, smoke-haze pollution, oxidative stress, non-small-cell lung cancer, transcriptomic profiling

## Abstract

Fine particulate matter (PM2.5) is a major air pollutant linked to lung cancer progression. In Southeast Asia, seasonal smoke-haze produces biomass-derived PM2.5, yet its acute effects on genetically diverse lung tumours remain unclear. We investigate how Chiang Mai haze-derived PM2.5 impacts oxidative stress and gene expression in three non-small-cell lung cancer (NSCLC) cell lines: A549 (*KRAS*-mutant), NCI-H1975 (*EGFR*-mutant), and NCI-H460 (*KRAS/PIK3CA*-mutant). Cells were exposed to PM2.5 (0–200 µg/mL) and assessed for viability (MTT), reactive oxygen species (ROS; H_2_O_2_, •OH) and malondialdehyde (MDA) levels, mitochondrial-associated fluorescence, and whole-transcriptome responses. Acute exposure caused dose- and time-dependent viability loss, with A549 and NCI-H1975 more sensitive than NCI-H460. ROS profiling normalized to viable cells revealed genotype-specific oxidative patterns: cumulative increases in A549, sharp reversible spikes in NCI-H1975, and modest changes in NCI-H460. MitoTracker intensity trended downward without significance, with subtle fluorescence changes and particulate uptake. RNA-seq identified robust induction of xenobiotic metabolism (*CYP1A1*, *CYP1B1*), oxidative/metabolic stress mediators (*GDF15*, *TIPARP*), and tumour-associated genes (*FOSB*, *VGF*), alongside repression of tumour suppressors (*FAT1*, *LINC00472*). Pathway enrichment analyses highlighted oxidative stress, IL-17, NF-κB, and immune checkpoint signaling. Together, biomass haze-derived PM2.5 from Northern Thailand drives genotype-dependent oxidative stress and transcriptional remodeling in NSCLC cells.

## 1. Introduction

Ambient air pollution remains one of the leading environmental threats to human health. According to the World Health Organization, almost the entire global population breathes air that exceeds WHO air quality guideline levels. Recurrent smoke-haze episodes in Southeast Asia, driven largely by biomass burning for agriculture and land clearing, generate airborne fine particulate matter (PM2.5, aerodynamic diameter ≤ 2.5 μm) with extremely high concentrations and distinct chemical compositions. PM2.5 is recognized as a major environmental carcinogen and global health burden. Understanding how such biomass-haze PM2.5 exposures are translated into cellular damage and cancer-related responses requires both accurate exposure characterization and detailed elucidation of molecular mechanisms of toxicity. Due to their large surface area and complex chemical composition, PM2.5 particles consist of various toxic compounds, including polycyclic aromatic hydrocarbons, trace metals, and other combustion-derived toxins [1]. PM2.5 particles can pass through the respiratory tract, penetrate deep into lung tissue, accumulate within the respiratory system, and induce oxidative stress along with triggering local inflammation that contributes to increased systemic inflammatory responses [2,3,4]. Epidemiological studies indicate that each 10 µg/m^3^ increase in long-term PM2.5 exposure is associated with an approximately 12% rise in lung-cancer mortality [5], with the strongest associations observed for non-small-cell lung cancer (NSCLC), which accounts for about 85% of all lung cancer cases [6]. However, the composition and concentration of PM2.5 vary across regions, shaped by local environmental factors, seasonal changes, and major emission sources [7,8,9]. In northern Thailand, for instance, the burning season shows that PM2.5 levels in rural areas were slightly higher than in urban centers, together with seasonal shifts in composition between both areas [7]. PM2.5 concentrations rise sharply during the dry season (March–April) in Northern Thailand, especially during smoke-haze episodes, often exceeding the WHO guideline levels. These peaks are mainly caused by biomass burning from agricultural residue and forest fires, and transboundary pollution, leading to a highly oxidizing chemical profile [8,9,10]. In Southeast Asia, recurrent smoke-haze episodes from biomass burning produce PM2.5 with high oxidative potential, yet its molecular effects on cancer cells remain poorly defined.

Reactive oxygen species (ROS), the highly reactive oxygen-based molecules, are central to cellular responses to environmental stress that can serve as a major signaling source and target. In cancer cells, ROS can increase proliferation, survival, angiogenesis, and metastasis. Nevertheless, excessive ROS accumulation causes oxidative stress that damages DNA, lipids, and proteins, drives genomic instability, alters the tumor microenvironment, promoting immune evasion, therapeutic resistance, and ultimately cell death [11,12,13]. Consequently, both ROS-reducing and ROS-enhancing strategies are being explored as adjunct treatments for NSCLC [14,15]. Mitochondria are generally recognized as a key source of ROS, and their status is often disturbed by environmental pollutants. Although PM2.5-induced mitochondrial dysfunction has been reported in airway epithelial models and animal studies [16], its impact on malignant cells, particularly genetically diverse NSCLC subtypes, remains unclear. Because NSCLC is metabolically heterogeneous, with *KRAS*- and *EGFR*-driven tumors showing distinct antioxidant capacities and bioenergetic demands, cellular redox homeostasis may represent a critical node of vulnerability to environmental carcinogens. Representative NSCLC models include A549 (*KRAS*-mutant), NCI-H1975 (*EGFR* L858R/T790M, *PIK3CA*-mutant, *TP53*-mutant), and NCI-H460 (*KRAS*/*PIK3CA*-mutant), each exhibiting different redox homeostasis and cellular profiles. Understanding how these subtypes respond to exogenous oxidative stress is essential, as redox adaptation not only determines sensitivity to environmental insults but also influences therapeutic resistance and metastatic potential [17].

Building on our previous preliminary data, in which we used an urban standard reference PM2.5 to define mutation-specific transcriptomic signatures in A549 and NCI-H1975 NSCLC cells [18], we now focus on real-world biomass-haze PM2.5 from Northern Thailand and integrate functional and transcriptomic readouts. In the present study, we extend the analysis to include a third NSCLC line (NCI-H460), quantify acute viability loss and oxidative stress (H_2_O_2_, •OH, MDA) across time, and combine these phenotypes with whole-transcriptome profiling and pathway enrichment. This integrated approach provides a more comprehensive, biomass-burning-specific perspective on how PM2.5 surges in smoke-haze episodes reshape redox balance and gene expression in genetically distinct NSCLC subtypes, aligning with the toxicological focus of the *Toxics* Special Issue on aerosol pollution from biomass burning.

Here, we investigate how PM2.5 collected during the seasonal smoke-haze in Chiang Mai, Northern Thailand, acutely affects oxidative stress and transcriptional regulation in NSCLC cells with distinct genetic backgrounds. Using viability assays, ROS profiling, mitochondrial imaging, and transcriptomic analysis, we demonstrate that biomass-derived PM2.5 triggers oxidative stress, and stress-response gene induction in a cell line-dependent manner. These findings provide mechanistic insight into how environmental carcinogens perturb tumor bioenergetics and suggest cellular ROS generation and redox pathways as key mediators of pollution-driven cancer progression.

## 2. Materials and Methods

### 2.1. PM2.5 Collection and Preparation

Chiang Mai seasonal PM2.5 samples were collected in Chiang Mai during smoke-haze season (March–April) in 2020 using a SA-1200 high-volume air sampler (Sierra Andersen, Smyrna, GA, USA) operating at 1130 L/min for 12 h per day (7:00 a.m. to 7:00 p.m.), drawing air through quartz-fiber filters (Whatman, UK, 8 × 10 inch) that had been pre-baked at 450 °C for 6 h. Filters were conditioned in a desiccator for 24 h prior to and after sampling to determine the PM2.5 mass, and the collected samples were stored at −20 °C until analysis [9]. For bulk preparation of the exposure material, pooled smoke-haze filters (total filter mass used ~2 g across multiple filters) were soaked in 500 mL deionized water for 24 h and sonicated in 30-min cycles in a water bath to extract the particles. Then, the suspension was vacuum-filtered (0.22 µm PVDF), lyophilized, and stored at −20 °C. For experiments, PM2.5 was resuspended in PBS at 5 mg/mL, sonicated for 30 min, and stored at −20 °C until use. Across the pooled filters, approximately 100 mg of dried PM2.5 extract was recovered and used for biological testing. Because particles were recovered by bulk aqueous extraction and processed by vacuum filtration (0.22 µm PVDF) prior to lyophilization, the tested material should be interpreted as the recovered aqueous PM2.5 extract (water-soluble and filter-passing fraction). Overall elution/recovery efficiency could not be quantified for the pooled bulk extraction, and hydrophobic/strongly filter-bound components (e.g., some black carbon-associated material) may be underrepresented, whereas water-soluble ions and water-soluble organics are expected to be retained through lyophilization.

### 2.2. Cell Lines and Culture Conditions

Human NSCLC cell lines, including lung adenocarcinoma (LUAD) lines A549 (ATCC CCL-185) and NCI-H1975 (ATCC CRL-5908), and the large cell carcinoma (LCC) line NCI-H460 (ATCC CRL-5801) were sourced from the American Type Culture Collection (ATCC, Manassas, VA, USA). Cells were cultured in RPMI-1640 medium (Gibco, Thermo Fisher Scientific, Waltham, MA, USA) supplemented with 10% fetal bovine serum (FBS), 1% L-glutamine, and 1% penicillin–streptomycin. Cultures were maintained at 37 °C in a 5% CO_2_ incubator (ESCO MEDICAL, Egå, Denmark) and routinely passaged when they reached 70–80% confluence.

### 2.3. Cell Viability (MTT) Assay

Cell viability after PM2.5 exposure was measured using the MTT assay. Cells were plated with 5 × 10^3^ cells per well in 96-well plates, with six replicates for each condition. After 24 h of incubation at 37 °C in 5% CO_2_, cells were treated with fresh medium containing PM2.5 at concentrations of 0 (control), 50, 100, or 200 μg/mL and then incubated for 24, 48 and 72 h. Following exposure, the medium was removed and replaced with 30 μL of MTT solution (2 mg/mL thiazolyl blue tetrazolium bromide; Sigma-Aldrich, St. Louis, MO, USA; Cat. No. M5655) per well and incubated for 4 h. Subsequently, 200 μL of dimethyl sulfoxide (DMSO) was added to each well to solubilize the formazan crystals. Absorbance was measured at 540 nm and 630 nm using SpectraMax iD3 microplate reader (Molecular Devices, San Jose, CA, USA). Cell viability was calculated as a percentage relative to the control group.

### 2.4. Intracellular Oxidative Stress Markers Measurement

To assess oxidative stress induced by PM2.5 exposure, three ROS-related markers, H_2_O_2_, •OH, and MDA, were measured using a SpectraMax iD3 microplate reader in 96-well plates, within biological triplicate with at least three technical replicates. Cells were seeded in 6-well plates at a density of 3 × 10^5^ cells/well and incubated at 37 °C in 5% CO_2_ for 24 h. After that, cells were treated with PM2.5 (0 or 200 μg/mL) for 24, 48, or 72 h, and harvested for each assay. For the H_2_O_2_ assay, cell pellets were resuspended in 1% (*w*/*v*) trichloroacetic acid (TCA), and the supernatant was mixed with 10 mM potassium phosphate buffer (pH 7.0) and potassium iodide. Absorbance was measured at 390 nm. For the •OH assay, pellets were homogenized in 20 mM potassium phosphate buffer (pH 6.0), and the supernatant was incubated with buffer containing 2-deoxy-D-ribose for 30 min at room temperature. After adding 0.5% (*w*/*v*) Thio barbituric acid (TBA) in 1.4% (*w*/*v*) TCA, samples were heated at 98 °C for 10 min, cooled to room temperature, and fluorescence was measured at 530 nm excitation and 600 nm emission. For MDA detection, pellets were resuspended in 0.1% (*w*/*v*) TCA, and the supernatant was reacted with 0.5% (*w*/*v*) TBA in 20% (*w*/*v*) TCA. Samples were incubated at 98 °C for 10 min, centrifuged at 10,000× *g* for 5 min, and absorbance was recorded at 532 and 600 nm.

### 2.5. Mitochondrial Status Measurement

Mitochondrial status was evaluated using MitoTracker™ Green FM (Invitrogen, Thermo Fisher Scientific, Waltham, MA, USA). Cells were seeded into 6-well plates at a density of 3 × 10^5^ cells per well and incubated at 37 °C in 5% CO_2_ for 24 h. After treatment with PM2.5 (0 or 200 μg/mL) for another 24 h, adherent cells were detached using 0.25% trypsin, collected, rinsed once with phosphate-buffered saline (PBS), and incubated with 500 nM MitoTracker™ Green FM for 30 min at 37 °C. Then, cells were washed twice with PBS containing 2% fetal bovine serum (FBS). Fluorescence was then measured using a microplate reader at 490 nm excitation and 530 nm emission.

### 2.6. Confocal Imaging

Confocal imaging was performed on cells prepared using the identical staining and harvesting protocol described in Section 2.5. To visualize mitochondrial and ROS localization after treatment, cells were incubated with either 500 nM MitoTracker™ Green FM or 20 μM H_2_DCFDA (Thermo Fisher Scientific) for 30 min at 37 °C, respectively. Following washing to remove excess dye, the single-cell suspension was transferred onto a standard glass slide with a coverslip, and then fluorescent images were acquired using a Leica Stellaris 5 confocal microscope (Leica Microsystems, Wetzlar, Germany) to capture images representing the morphology and fluorophore localization within single cells in suspension. Fluorescence intensity was quantified using LAS X (v5.3.1) software.

### 2.7. RNA Isolation and Sequencing

Total RNA was extracted from the three NSCLC cell lines—A549, NCI-H1975, and NCI-H460—following the PM2.5 exposure or control treatment. Cells were initially seeded in 6-well plates at a density of 3 × 10^5^ cells/well and incubated at 37 °C in 5% CO_2_ for 24 h. Following this pre-incubation, cells were treated with 200 μg/mL biomass haze-derived PM2.5 or with vehicle control for an additional 24 h. After that, cells were harvested and RNA was immediately extracted using the Direct-zol™ RNA Miniprep kit (Zymo Research, Irvine, CA, USA; Cat. No. R2050), which includes a DNase step to remove contaminating DNA. RNA quality was evaluated to ensure compatibility with next-generation sequencing (NGS), with each sample required to meet the following criteria: a minimum volume of 50 µL, concentration of at least 50 ng/µL, an OD 260/280 ratio near 2.0, and intact RNA confirmed by gel electrophoresis. Samples that passed these thresholds were used for library construction, which included mRNA capture, fragmentation, cDNA synthesis, end polishing, adapter ligation, and PCR amplification. Sequencing was performed using the Illumina NovaSeq 6000 system (Illumina, San Diego, CA, USA), producing 150 bp paired end reads with a target depth of approximately 30 million reads per sample to ensure transcriptome coverage. RNA-seq was performed using three biological replicates per condition for each cell line.

### 2.8. Transcriptomic Analysis

Raw reads were quality-checked using FastQC (v0.12.1) and adapter/low-quality bases were trimmed using Trimmomatic (v0.40). Trimmed reads were aligned to the human reference genome (GRCh38) using HISAT2 (v2.2.1). Gene-level read counts were generated using featureCounts (v2.1.1) with GENCODE annotation. Differential expression analysis was performed in DESeq2 (v1.50.2) using raw count matrices (false discovery rate (FDR) < 0.05; |log2FC| ≥ 1). Functional enrichment analyses were conducted using clusterProfiler (v4.18.4) against KEGG and Gene Ontology (GO) databases with multiple-testing correction.

### 2.9. Statistical Analysis

All results are expressed as mean ± SD from at least three independent experiments. A *p*-value of ≤0.05 was considered statistically significant. Two-way ANOVA with Tukey’s post hoc test was used for viability assays, and unpaired *t*-tests were used for ROS/MDA and MitoTracker Green fluorescence, as indicated in figure legends. For transcriptomic data, differential expression was assessed using DESeq2 as described above, with an adjusted *p*-value (FDR) < 0.05 and |log2 fold change| ≥ 1.0 considered significant.

## 3. Results

### 3.1. PM2.5 Reduces NSCLC Cell Viability in a Genetic Background-Dependent Manner

**Figure 1:**
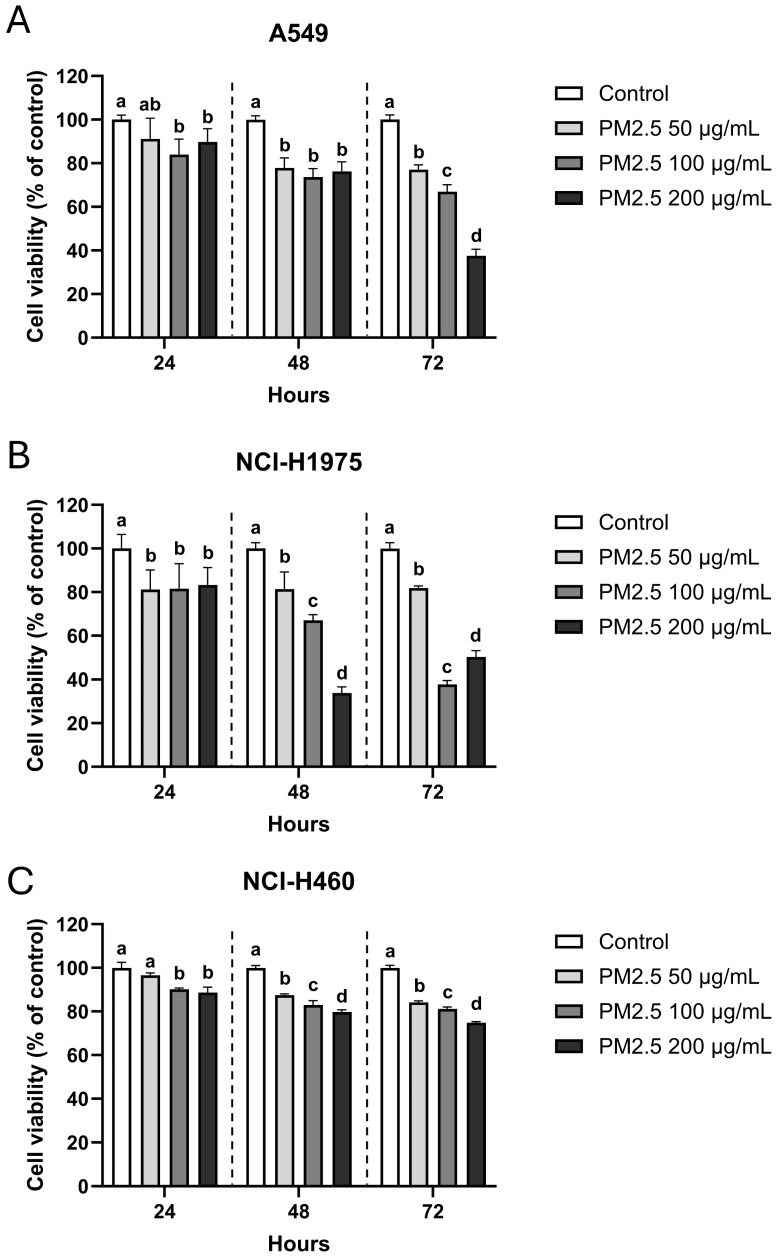
Dose- and time-dependent reduction of NSCLC cell viability after PM2.5 exposure. (**A**) A549, (**B**) NCI-H1975, and (**C**) NCI-H460 cells were exposed to 0 (vehicle control), 50, 100, and 200 µg/mL PM2.5 for 24, 48, and 72 h. Cell viability was measured by MTT assay and expressed as percentage of untreated control. Data are presented as mean ± SD from six replicates. Within each time point, groups sharing the same letter are not significantly different, whereas groups with different letters differ significantly (*p* < 0.05).

MTT assays demonstrated a clear dose- and time-dependent reduction in viability following exposure to biomass smoke-derived PM2.5 in all three NSCLC cell lines, though with distinct sensitivities. A549 (*KRAS*-mutant) cells were the most vulnerable, with viability decreasing steadily over time and dropping to 37.6 ± 2.7% at 200 µg/mL after 72 h, compared to 66.9 ± 2.9% and 77.1 ± 2.0% at 100 and 50 µg/mL, respectively (Figure 1A). NCI-H1975 (*EGFR* L858R/T790M, *PIK3CA*- and *TP53*-mutant) cells exhibited a similar but more acute pattern (Figure 1B): viability fell sharply at 48 h (33.8 ± 2.6% at 200 µg/mL), with partial recovery by 72 h (50.2 ± 2.6%). By contrast, NCI-H460 (*KRAS* and *PIK3CA*-mutant) cells displayed relative resistance, maintaining >70% viability across all concentrations and time points, including 74.8 ± 0.5% at the highest dose after 72 h (Figure 1C). These divergent responses suggest that genetic background critically influences cellular susceptibility to PM2.5.

### 3.2. PM2.5 Induces ROS Accumulation and Lipid Peroxidation in NSCLC Cells

**Figure 2:**
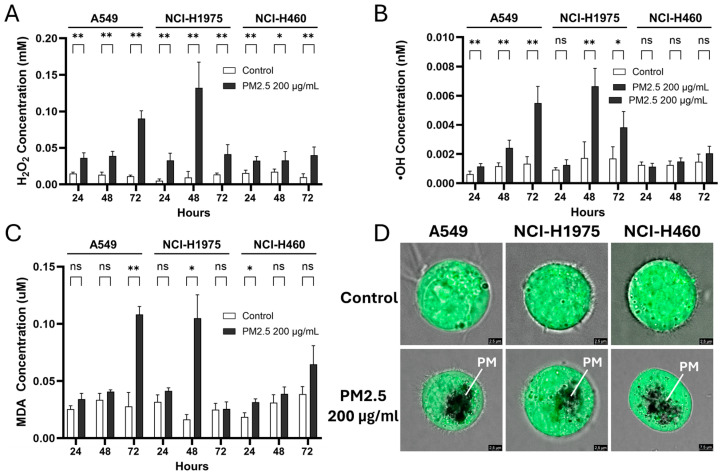
Biomass haze-derived PM2.5 induces ROS accumulation and oxidative damage in NSCLC cells. Relative levels of (**A**) hydrogen peroxide (H_2_O_2_), (**B**) hydroxyl radicals (•OH), and (**C**) malondialdehyde (MDA) were measured in control cells and cells exposed to 200 µg/mL PM2.5 for 24, 48, and 72 h. Values were normalized to cell viability. (**D**) Representative H_2_DCFDA fluorescence images show intracellular ROS staining; PM indicates particulate matter (PM2.5) visualized within the cells. Data are presented as mean ± SD from at least three independent experiments. Statistical significance (determined by unpaired *t*-tests): * *p* < 0.05, ** *p* < 0.01; ns = not significant.

PM2.5 exposure caused a time-dependent increase in ROS in all NSCLC cell lines, though with distinct kinetics (Figure 2A–C). Hydrogen peroxide (H_2_O_2_) levels rose steadily in A549 cells, reaching 0.090 ± 0.010 mM at 72 h. In NCI-H1975 cells, H_2_O_2_ exhibited a sharp transient surge at 48 h (0.132 ± 0.032 mM) that declined by 72 h, whereas NCI-H460 showed only modest but consistent elevations at all time points (0.033–0.040 mM) (Figure 2A). Hydroxyl radical (•OH) accumulation displayed similar genotype-specific trends (Figure 2B). A549 cells showed progressive increases, more than doubling by 72 h (0.0055 ± 0.0010 nM), while NCI-H1975 cells exhibited a delayed but dramatic spike at 48 h (0.0066 ± 0.0011 nM) followed by a reduction at 72 h. In NCI-H460 cells, no significant changes in •OH were detected. Lipid peroxidation, measured as malondialdehyde (MDA), further highlighted these divergent stress responses. A549 cells showed a late but substantial rise at 72 h (0.114 ± 0.006 mM), NCI-H1975 cells exhibited a transient peak at 48 h (0.116 ± 0.020 mM), and NCI-H460 displayed only a modest, early increase at 24 h that was not sustained (Figure 2C). Fluorescence imaging using H_2_DCFDA, a ROS-specific probe, confirmed intracellular ROS accumulation and visible particulate uptake (Figure 2D).

Together, these results demonstrate that biomass haze-derived PM2.5 strongly induces ROS production and oxidative damage in a cell line-dependent manner. Elevated H_2_O_2_ and •OH may disrupt cellular bioenergetics and contribute to reduced viability, implicating oxidative stress as a key driver of cytotoxicity in susceptible subtypes. However, lipid peroxidation responses were less consistent.

### 3.3. Effect of PM2.5 on Mitochondrial-Associated Fluorescence

**Figure 3:**
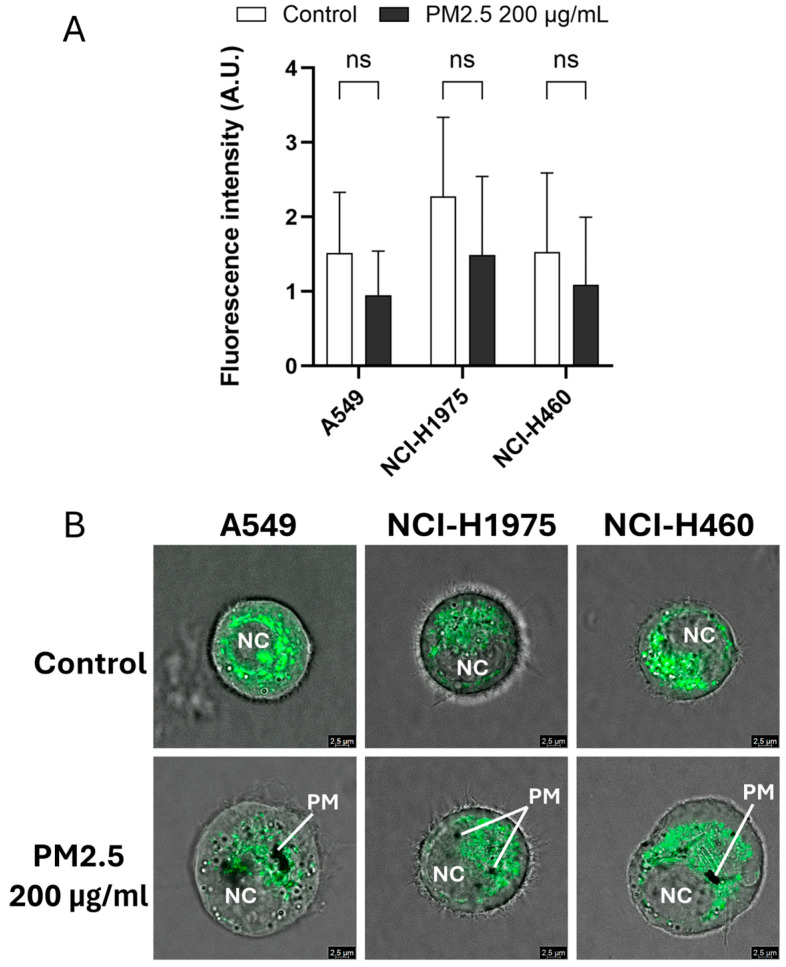
Biomass haze-derived PM2.5 alters mitochondrial fluorescence patterns in NSCLC cells. (**A**) Quantification of MitoTracker™ Green FM fluorescence intensity in A549, NCI-H1975, and NCI-H460 cells after 24 h exposure to 200 µg/mL PM2.5. (**B**) Representative confocal images show mitochondrial fluorescence in PM2.5-treated cells. PM: particulate matter; NC: nucleus. Data are presented as mean ± SD from three independent experiments. Statistical significance (determined by unpaired *t*-tests): ns = not significant.

Mitochondrial status was assessed using MitoTracker™ Green FM fluorescence after exposure to biomass haze-derived PM2.5. Across all NSCLC cell lines, a trend toward reduced fluorescence intensity was observed following 24 h treatment with 200 µg/mL PM2.5, although differences did not reach statistical significance (Figure 3A). This decrease suggests potential alterations in mitochondrial content or dye uptake efficiency under stress conditions.

Confocal imaging provided further spatial insight into mitochondrial fluorescence patterns within single suspended cells (Figure 3B). Control cells exhibited bright, filamentous mitochondrial networks, whereas PM2.5-treated cells displayed weaker and more diffuse staining, accompanied by visible intracellular particulate deposits. Given the qualitative nature of these images and the limitations of MitoTracker™ Green for measuring mitochondrial membrane potential or ROS directly, we interpret these data as indicative of subtle perturbations in mitochondrial-associated fluorescence under acute oxidative stress, rather than definitive evidence of mitochondrial dysfunction. More specialized probes and single-cell imaging or flow cytometry will be required in future studies to directly quantify mitochondrial ROS production and bioenergetic impairment after biomass-haze PM2.5 exposure.

### 3.4. Biomass Haze-Derived PM2.5 Alters Gene Expression Patterns in NSCLC Cells

**Figure 4:**
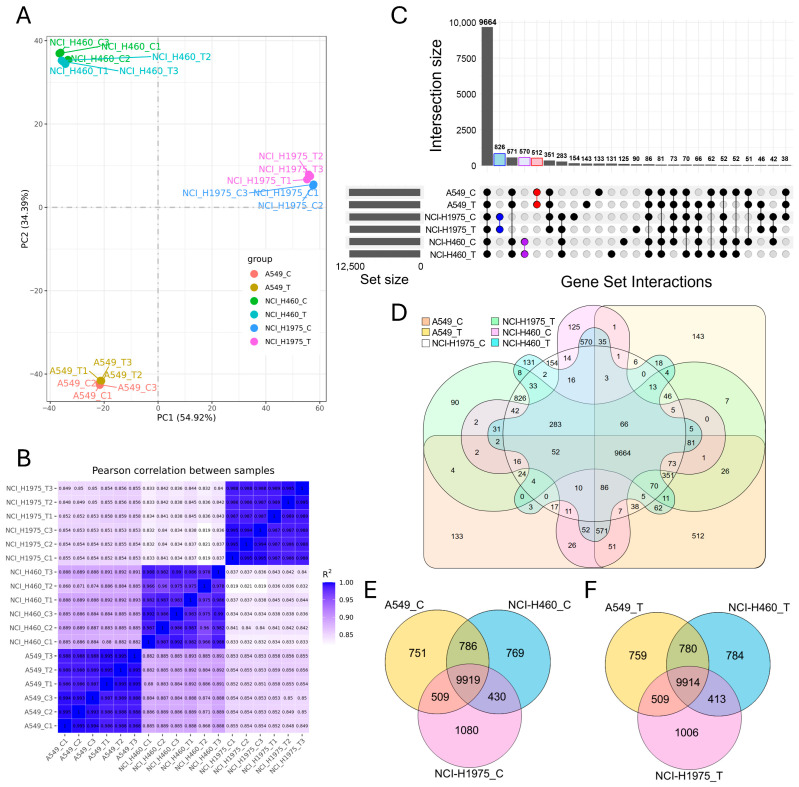
Global transcriptomic profiling of NSCLC cell lines exposed to biomass haze-derived PM2.5. (**A**) Principal component analysis (PCA) reveals clear segregation of A549, NCI-H1975, and NCI-H460 transcriptomes, with separation between control (**C**) and PM2.5-treated (T) groups within each line. (**B**) Pearson correlation heatmap confirms high intra-group reproducibility (R^2^ ≥ 0.95) and highlights divergence between genotypes and treatment conditions. (**C**) UpSet plot and (**D**) six-way Venn diagram illustrate both the large conserved core transcriptome and condition-specific gene sets across all experimental groups. (**E**,**F**) Three-way Venn diagrams compare transcriptomes across A549, NCI-H1975, and NCI-H460 under control (**E**) and PM2.5 treatment (**F**), showing a stable shared core alongside cell line-specific expression signatures.

Global transcriptomic analysis demonstrated that biomass haze-derived PM2.5 exposure reshaped gene expression in all NSCLC cell lines in a cell type-dependent manner (Figure 4). Principal component analysis (PCA) revealed that samples clustered primarily by cell line identity, yet within each line, PM2.5-treated groups separated from controls (Figure 4A). Pearson correlation confirmed strong intra-group reproducibility (R^2^ ≥ 0.95) and highlighted transcriptional divergence between both different cell types and treatment conditions (Figure 4B).

Intersection analysis using UpSet plots and Venn diagrams revealed both a large conserved core and condition-specific expression changes. Across all samples, 9664 genes were commonly expressed (Figure 4C,D). Within each cell line, substantial overlap was observed between control and exposed groups, including 512 shared genes in A549, 826 in NCI-H1975, and 570 in NCI-H460. Nevertheless, each condition also displayed unique expression profiles, with 133 genes specific to A549 control, 143 to A549 exposed, 154 to NCI-H1975 control, 90 to NCI-H1975 exposed, 125 to NCI-H460 control, and 131 to NCI-H460 exposed.

Three-way Venn diagrams comparing cell lines under control (Figure 4E) and PM2.5-exposed conditions (Figure 4F) highlighted a highly conserved transcriptome across all lines, with 9919 and 9914 shared genes, respectively. However, each line also retained distinct sets of uniquely expressed transcripts, reflecting genotype-driven transcriptional programs. Notably, PM2.5 exposure appeared to accentuate these differences, with an increase in condition-specific transcripts particularly evident in NCI-H1975, consistent with its acute oxidative stress phenotype observed in viability and ROS assays. Together, these results indicate that acute PM2.5 exposure preserves a stable core transcriptome across NSCLC cells but simultaneously triggers lineage- and mutation-specific transcriptional remodelling. This dual pattern underscores how environmental stress imposes both a universal redox response and divergent, genotype-dependent adaptations.

### 3.5. Differential Expression Analysis Suggests a Core PM2.5-Responsive Stress Program with Lineage-Specific Magnitude

**Figure 5:**
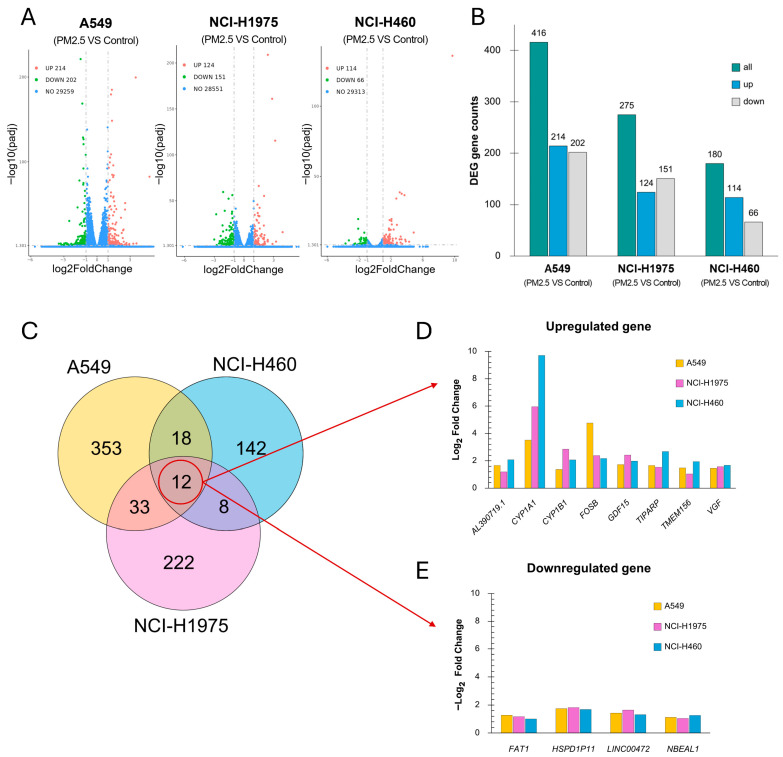
Differential gene expression profiles in NSCLC cell lines exposed to biomass haze-derived PM2.5. (**A**) Volcano plots showing significantly upregulated (green) and downregulated (red) genes for each cell line when comparing control and PM2.5 treatment (adjusted *p* ≤ 0.05, |log_2_FC| ≥ 1.0). (**B**) Bar chart summarizing the number of differentially expressed genes (DEGs) (green) of each cell line, separated into upregulated (blue) and downregulated (grey) genes. (**C**) Venn diagram illustrating the overlap of DEGs across the three cell lines. (**D**,**E**) Log_2_ fold changes for commonly upregulated genes (**D**) or downregulated (**E**) genes across cell lines, with colors indicating each cell identity.

Differential expression analysis demonstrated that biomass haze-derived PM2.5 exposure elicited distinct transcriptional reprogramming across NSCLC cell lines (Figure 5). Volcano plots showed numerous genes significantly altered under treatment (adjusted *p*-value ≤ 0.05 and |log_2_ fold change| ≥ 1.0), with A549 exhibiting the largest DEG set (214 upregulated, 202 downregulated), followed by NCI-H1975 (124 up, 151 down) and NCI-H460 (114 up, 66 down) (Figure 5A,B).

Venn analysis identified 12 genes consistently affected across all three models (8 upregulated, 4 downregulated) (Figure 5C). Among the commonly upregulated genes (Figure 5D), *CYP1A1* and *CYP1B1*, central to xenobiotic metabolism and oxidative detoxification, showed robust induction across all lines. *CYP1A1* showed the strongest induction, with log_2_ fold changes of 9.71 in NCI-H460, 5.96 in NCI-H1975, and 3.51 in A549. *CYP1B1* was also elevated across the lines (NCI-H460: 2.07; NCI-H1975: 2.86; A549: 1.36). *GDF15*, which plays a role in cellular stress responses and tumor progression, was upregulated with log_2_ fold changes of 1.97 in NCI-H460, 2.42 in NCI-H1975, and 1.72 in A549. *FOSB* and *TIPARP*, regulators of stress adaptation and DNA repair, were also elevated, with the strongest *FOSB* induction in A549 (4.76) and highest *TIPARP* induction in NCI-H460 (2.67). Additional shared stress-response genes, including *VGF*, *TMEM156*, and *AL390719.1*, exhibited moderate but consistent upregulation.

The commonly downregulated genes (Figure 5E) included *FAT1* and *LINC00472,* both implicated in tumor suppression through regulation of cell adhesion, migration, and apoptosis. Their suppression was observed across all lines (average log_2_FC range −1.0 to −1.6). Two additional transcripts, *HSPD1P1* (a pseudogene related to mitochondrial chaperonin HSPD1) and *NBEAL1*, were also consistently reduced, though their functional roles remain unclear. Collectively, these findings indicate that PM2.5 exposure induces a conserved set of xenobiotic metabolism and stress-response genes, coupled with suppression of tumor-suppressor pathways. The magnitude of induction varied by genotype, with NCI-H460 showing the strongest xenobiotic gene activation, A549 demonstrating pronounced stress transcription factor responses, and NCI-H1975 exhibiting intermediate but broad changes. This pattern underscores the heterogeneity of NSCLC redox adaptation to environmental particulate stress.

### 3.6. Pathway Enrichment Analysis Reveals Shared Oxidative and Immune Stress Responses with Lineage-Specific Adaptations

**Figure 6:**
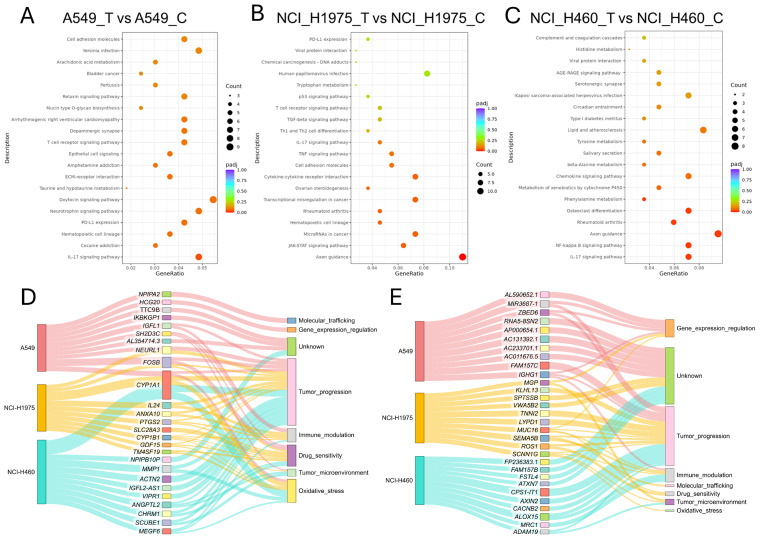
Functional and pathway enrichment analysis of differentially expressed genes (DEGs) in NSCLC cells exposed to biomass haze-derived PM2.5. (**A**–**C**) Dot plots of KEGG pathway enrichment for DEGs in A549 (**A**), NCI-H1975 (**B**), and NCI-H460 (**C**) cell lines. The gene ratio reflects the proportion of DEGs involved in each pathway, and bubble size corresponds to the number of genes. (**D**,**E**) Sankey diagrams depicting the categorized biological functions of the top 10 upregulated (**D**) and downregulated (**E**) genes in each cell line.

KEGG pathway enrichment analysis of differentially expressed genes (DEGs) identified both conserved and cell line-specific pathways activated by biomass haze-derived PM2.5 exposure (Figure 6A–C). Across all models, the IL-17 signaling pathway was consistently enriched, reflecting a common pro-inflammatory response. In the lung adenocarcinoma (LUAD) cell lines A549 and NCI-H1975, additional enrichment was observed for pathways linked to PD-L1/PD-1 immune checkpoint regulation, T-cell receptor signaling, and adhesion molecules, suggesting activation of immune evasion and tumor–immune interactions. A549 cells exhibited broader enrichment, including arachidonic acid metabolism and ECM–receptor interactions, pathways closely tied to ROS signaling and extracellular stress adaptation. NCI-H1975 cells displayed a more immune-focused profile, with strong enrichment of JAK-STAT, TNF, and TGF-β signaling pathways, consistent with their acute ROS phenotype and activation of DNA damage and inflammatory cascades. In contrast, NCI-H460 (large-cell carcinoma) cells showed enrichment for NF-κB and chemokine signaling pathways, along with xenobiotic metabolism by cytochrome P450, supporting their relative resistance through enhanced detoxification capacity.

Functional categorization of the top 10 upregulated genes in each line further highlighted these divergent responses (Figure 6D). Genes clustered predominantly into categories of tumor progression, oxidative stress response, immune modulation, and drug sensitivity. Notably, *CYP1A1* was among the most strongly upregulated transcripts across all cell lines, consistent with activation of xenobiotic metabolism, linking PM2.5 exposure to both oxidative stress adaptation and tumor-promoting processes. In A549, upregulated genes were distributed across all eight categories, reflecting broad pathway activation, while NCI-H1975 showed concentration in tumour progression and immune modulation. NCI-H460 upregulated genes were primarily associated with tumour progression and detoxification, aligning with their resistant phenotype.

Analysis of the top 10 downregulated genes (Figure 6E) showed predominant clustering in tumour progression, immune modulation, and gene expression regulation, with additional genes of unknown function. In A549, downregulated genes were enriched for transcriptional regulators, suggesting disruption of gene expression control. NCI-H1975 displayed the broadest distribution, spanning all eight categories, including oxidative stress and molecular trafficking, while NCI-H460 was dominated by suppression of tumour progression and immune-related genes. Together, these enrichment analyses reveal that PM2.5 exposure drives a dual program of shared oxidative stress and inflammatory signalling, while the scale and nature of responses differ by genotype. LUAD cells (A549, NCI-H1975) preferentially activated immune and checkpoint pathways, while NCI-H460 favoured detoxification and stress-buffering mechanisms. This underscores the heterogeneity of NSCLC adaptation to environmental carcinogens.

## 4. Discussion

### 4.1. Cell Line-Specific Responses Shaped by Genetic Background

This study examined the acute effects of Chiang Mai haze-derived PM2.5 on three NSCLC cell lines: A549 and NCI-H1975 (LUAD) and NCI-H460 (LCC). A relatively high concentration of PM2.5 (200 µg/mL, 24 h) was used, which exceeds typical environmental levels but is consistent with in vitro models designed to simulate acute high-exposure events. Similar in vitro concentrations (10–400 µg/mL) have been widely applied to reveal both long and short-term effects of PM2.5 that can reduce cell survival and induce oxidative stress, DNA damage, mitochondrial dysfunction, and inflammatory signaling [19,20,21,22,23]. These findings indicate that acute PM2.5 exposure can rapidly induce oxidative and genotoxic stress, providing a relevant model for studying early molecular responses in NSCLC progression. Importantly, our data revealed distinct cellular phenotypes shaped by the genetic background of each line, even between LUAD subtypes, A549 and NCI-H1975.

NCI-H1975 cells (*EGFR*, *PIK3CA*, and *TP53* mutant; *KRAS* wild-type) showed the strongest acute oxidative burst, with sharp increases in H_2_O_2_ and •OH at 48 h, accompanied by reduced viability and transient lipid peroxidation. Partial recovery by 72 h suggests activation of compensatory pathways, consistent with reports that *EGFR*-mutant NSCLC cells are hypersensitive to redox imbalance but capable of adaptive signaling [24,25]. On the other hand, A549 cells (*KRAS*-mutant; *EGFR*, *PIK3CA*, and *TP53* wild-type) showed a slower but more sustained response. ROS levels climbed steadily and reached their peak at 72 h, along with a substantial increase in MDA, pointing to cumulative oxidative damage and compromised membrane integrity. Viability steadily declined without recovery, reflecting persistent stress and a cytotoxic response rather than an acute but reversible insult, as seen in NCI-H1975. This pattern indicates persistent bioenergetic stress and aligns with studies reporting PM2.5-driven apoptosis and oxidative damage in A549 models [19,20,22,23]. NCI-H460 cells—*PIK3CA* and *KRAS*-mutant, *EGFR* and *TP53* wild-type, LCC cell line, demonstrated relative resistance. Viability was most maintained, ROS increases were modest, and lipid peroxidation remained minimal. This resilience may reflect stronger antioxidant defenses or metabolic adaptations that buffer against PM2.5-induced stress.

Together, these results highlight the heterogeneity of NSCLC responses to particulate matter, even within LUAD subtypes. The contrast between acute but partly reversible stress in NCI-H1975, cumulative oxidative burden in A549, and resilience in NCI-H460 underscores how tumor genotype and lineage influence redox vulnerability to environmental insults.

### 4.2. Chiang Mai PM2.5 Composition and Acute ROS Surge

The PM2.5 used in this study was collected during the 2020 smoke-haze episode in Chiang Mai, a period dominated by biomass combustion [8]. Acute exposure of NSCLC cells to these materials induced a rapid increase in H_2_O_2_, •OH, and MDA. The magnitude and timing of these effects were strongly genotype-dependent: A549 cells accumulated ROS and lipid peroxidation progressively, NCI-H1975 cells experienced transient oxidative spikes with partial recovery, and NCI-H460 cells mounted only a modest response.

Chemical characterization of the collected particles confirmed a biomass-burning signature, with organic carbon (OC) as the dominant component. Among these, levoglucosan, a tracer of cellulose pyrolysis, served as a clear indicator of biomass combustion [9]. Another major class of compounds, polycyclic aromatic hydrocarbons (PAHs), are metabolized by cytochrome P450 enzymes (*CYP1A1*, *CYP1A2*, *CYP1B1*), producing reactive intermediates that can initiate carcinogenesis and engage in redox cycling, thereby amplifying ROS generation [26]. In addition, a previous study in Chiang Mai reported that PM2.5-bound fatty acids, particularly during biomass-burning periods, can influence the strong oxidative potential, underscoring the contribution of organic aerosols to ROS formation [27].

Importantly, the toxicity of PM2.5 is source-dependent. A recent study reported that H460 cells were more sensitive to PM2.5-induced apoptosis at lower concentrations, while A549 cells required higher doses for comparable effects; both, however, exhibited enhanced migration following exposure [28]. Several comparative studies show that PM2.5 derived from biomass burning often exhibits greater oxidative potential than urban PM when normalized per unit mass, due to abundant carbonaceous organic compounds and redox-active organics (e.g., PAHs, quinones) [29]. In contrast, urban PM frequently contains higher loads of transition metals (Fe, Cu, Mn) and nitrate or sulfate salts, which promote Fenton reactions and rapid generation of hydroxyl radicals upon entry to cells [30]. Kim et al. (2025) also report that oxidative potential (OP) of urban PM2.5 correlates strongly with metal content and secondary species, and less with bulk mass, highlighting compositional effects beyond simple concentration metrics [31]. Epidemiological source-apportionment studies further support this distinction, showing that biomass burning can dominate PM2.5 mass while fossil-fuel combustion sources may account for a disproportionate share of cardiovascular risk, emphasizing that toxicity cannot be inferred from mass alone.

For comparison with the earlier urban-reference dataset using the NIST standard particulate matter SRM 1648a [18], we highlight key differences and shared outcomes. The SRM 1648a study used RNA-seq in A549 and NCI-H1975 cells following acute exposure and identified mutation-dependent oxidative stress and xenobiotic-response programs. In the present work, we apply the same comparative framework to locally collected Chiang Mai smoke-haze PM2.5 (biomass-burning season, March–April 2020) and broaden the experimental readouts to include dose/time-resolved cytotoxicity, oxidative stress markers (H_2_O_2_, •OH, MDA), and mitochondrial imaging, while adding NCI-H460 to capture inter-line variability. Across both datasets, responses are strongly lineage- and mutation-dependent, with prominent remodeling in EGFR-mutant NCI-H1975 cells [18,24]; SRM 1648a therefore serves as an urban benchmark, whereas our haze-derived dataset links transcriptomic signatures to oxidative phenotypes, albeit without a matched non-haze urban comparator.

Composition likely contributes to these patterns. Urban PM2.5 is often relatively enriched in transition metals that promote rapid radical generation via Fenton-type chemistry, whereas haze PM2.5 is biomass-dominant and likely more influenced by organic constituents that drive xenobiotic metabolism and redox cycling, sustaining intracellular ROS. This framing aligns with SRM 1648a transcriptomic responses [18,24] and with lower carbon/nitrogen in SRM 1648a than some urban PM2.5, suggesting reduced organic loading despite broadly similar elemental features [32]. Finally, because Chiang Mai PM2.5 was prepared by pooled bulk aqueous extraction and 0.22 µm filtration and recovery was not quantified, exposures should be interpreted as an operationally defined aqueous PM2.5 extract, consistent with prior Chiang Mai biomass PM2.5 protocols [33].

These discrepancies emphasize that both aerosol chemical composition and cell-intrinsic defenses shape the trajectory of oxidative stress. In our study, Chiang Mai biomass-derived PM2.5, enriched in organic carbons including PAHs, produced a profile characterized by enhanced ROS generation and lipid peroxidation, revealing a strong link between particle chemistry, cellular redox vulnerability, and tumor cell heterogeneity.

### 4.3. ROS-Driven Stress Signaling and Transcriptomic Reprogramming

Our data indicate that acute exposure to biomass haze-derived PM2.5 primarily perturbs NSCLC cells through robust intracellular ROS generation, with mitochondria likely acting as both sources and targets within this stress network. Bulk assays demonstrated marked increases in H_2_O_2_, •OH, and MDA in A549 and NCI-H1975 cells, consistent with oxidative damage to lipids and other macromolecules, while NCI-H460 displayed more modest changes. MitoTracker Green measurements and confocal imaging suggested a trend toward reduced mitochondrial-associated fluorescence and altered staining patterns after PM2.5 exposure, but these changes were not statistically significant and cannot be equated with overt mitochondrial dysfunction. Instead, we interpret the mitochondrial readouts as qualitatively consistent with a broader redox imbalance induced by biomass-haze PM2.5, while recognizing that direct assessment of mitochondrial membrane potential and mitochondrial ROS will require more specific probes and single-cell approaches in future work.

Transcriptomic profiling based on RNA-seq analysis revealed strong upregulation of xenobiotic-metabolizing genes, particularly *CYP1A1* and *CYP1B1*, both members of the cytochrome P450 enzyme family and transcriptionally regulated by the aryl hydrocarbon receptor (AhR) upon PAHs activation. While these enzymes detoxify xenobiotics, their metabolic activity also generates reactive intermediates that amplify oxidative stress, DNA adduct formation, and carcinogenic risk [34,35,36]. These findings are consistent with previous studies that reported upregulation of *CYP1A1* and *CYP1B1* after PM2.5 exposure in LUAD, human bronchial epithelial cell lines, and vascular smooth muscle cells [24,37,38]. Recent work also noted cell type-specific differences, with PM2.5 driving AhR-dependent *CYP1A1* but not *CYP1B1* in nasal epithelial cells [39]. This suggests nuanced tissue-specific regulation of AhR signaling.

Other stress-responsive genes were also consistently induced. *GDF15*, a cytokine linked to oxidative and metabolic stress, is elevated in damaged tissues and promotes adaptive signaling [40,41]. *FOSB*, an AP-1 family transcription factor, has dual context-dependent roles: promoting tumorigenesis in p53-null cells but activating tumor-suppressive programs in wild-type p53 backgrounds [42]. In A549 (*TP53* wild-type), strong *FOSB* induction likely reflects an attempted stress-adaptive response, though insufficient to prevent cytotoxicity. *TIPARP*, a negative feedback regulator of AhR, was most strongly induced in NCI-H460, suggesting a protective mechanism limiting sustained *CYP1A1* activation and thereby buffering against excessive ROS [43,44]. Additionally, the upregulation of VGF, a neuropeptide gene known to protect neurons from stress-induced apoptosis, is consistent with reports that it confers resistance to EGFR-TKI therapy in LUAD [45]. In contrast, we observed downregulation of *FAT1* and *LINC00472*, both of which function as tumour suppressors. *FAT1* reduction has been shown to promote epithelial–mesenchymal transition and activate signaling pathways associated with cancer initiation and progression in multiple cancer types [46,47], while the increase in *LINC00472* levels suppressed proliferation, motility, and tumor progression by inducing expressions of tumor suppressor genes and decreasing the expressions of oncogenes [48,49].

Genotype-specific stress signatures provide further mechanistic insight. NCI-H460 cells displayed the strongest induction of *CYP1A1*, a canonical AhR target, together with *TIPARP*, a negative regulator of AhR signaling. This dual activation suggests a feedback-protected xenobiotic metabolism program: while *CYP1A1* drives detoxification and ROS generation, *TIPARP* limits sustained AhR activation, helping explain the relative resistance of H460 cells to PM2.5 cytotoxicity. In contrast, A549 cells, which accumulated ROS progressively and showed continuous viability loss, exhibited the highest induction of *FOSB*. Given their wild-type *TP53* status, *FOSB* upregulation likely reflects a p53-dependent stress-adaptive response; however, the persistent cytotoxicity indicates this compensation was inadequate, marking *FOSB* as a stress marker rather than an effective defense. NCI-H1975 cells demonstrated sharp ROS spikes with partial recovery, accompanied by strong induction of *GDF15*, *CYP1A1*, and *CYP1B1*. This combination reflects substantial oxidative and metabolic stress (*GDF15*) coupled with xenobiotic metabolism that both detoxifies and amplifies ROS, producing a dual response of injury and survival that may underline their transient recovery phenotype.

Pathway enrichment analysis reinforced these differences. A shared response was activation of IL-17 signaling, a pathway linking ROS to chronic inflammation and cancer progression [50]. In LUAD models, PM2.5 exposure enhanced immune checkpoint and cytokine signaling, suggesting ROS-driven remodeling of the tumor–immune interface. These observations align with multi-cohort studies linking ROS-related signatures in LUAD to impaired dendritic-cell activation and antitumor immunity [51]. By contrast, NCI-H460 relied more on NF-κB, chemokine, and xenobiotic metabolism pathways, underscoring the tight connection between oxidative stress and inflammatory reprogramming [16,52].

**Figure 7:**
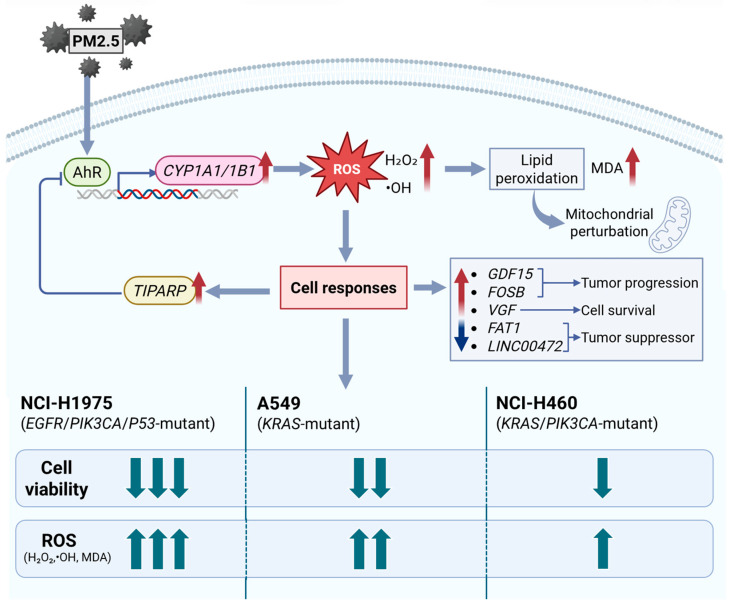
Proposed mechanism of oxidative and transcriptional responses to biomass haze-derived PM2.5 in NSCLC cells. PM2.5 exposure activates the AhR signaling cascade, inducing *CYP1A1/1B1* expression and ROS generation (H_2_O_2_, •OH). Accumulated ROS causes lipid peroxidation (measured as MDA), driving the activation of stress- and tumor-associated genes (*GDF15*, *FOSB*, *VGF*) and suppression of tumor suppressors (*FAT1*, *LINC00472*). *TIPARP* provides negative feedback on AhR activity. Upward arrows indicate increased/upregulated; downward arrows indicate decreased/downregulated. Comparative responses show NCI-H1975 > A549 > NCI-H460 in oxidative and cytotoxic intensity, reflecting genotype-specific vulnerabilities to environmental particulate exposure.

Figure 7 proposes the mechanism on how biomass haze-derived PM2.5 from Northern Thailand provokes oxidative stress and gene expression changes in three NSCLC cells. When exposed to PM2.5, PAHs activate the AhR pathway, which in turn stimulates the expression of the xenobiotic-metabolizing genes *CYP1A1* and *CYP1B1*. While these genes normally help detoxify harmful compounds, their activity could also potentially produce ROS, including H_2_O_2_ and •OH, damaging cellular membranes and inducing mitochondrial perturbation. This leads to lipid peroxidation, as measured by increased MDA levels. This then triggers distinct transcriptional responses, with *GDF15* and *FOSB* linked to stress adaptation and tumor progression, *VGF* supporting cell survival, and *TIPARP* acting as a feedback regulator to moderate AhR activity. Meanwhile, tumor suppressors *FAT1* and *LINC00472* are suppressed, suggesting loss of adhesion and growth control. Among the three cell models, NCI-H1975 (*EGFR/PIK3CA/TP53* mutant) experiences the sharpest transient rise in ROS with partial recovery of viability, A549 (*KRAS* mutant) shows the most sustained oxidative burden and greatest cumulative loss of viability, and NCI-H460 (*KRAS/PIK3CA* mutant) remains comparatively resilient with strong TIPARP induction. Overall, the schematic shows how Chiang Mai haze-derived PM2.5 induces oxidative damage and unique stress signatures in NSCLC cells, emphasizing that genetic background shapes how tumors sense and adapt to environmental particulate exposure.

### 4.4. Clinical and Public Health Implications of Biomass Haze PM2.5 in NSCLC

Our findings suggest that Chiang Mai haze is not just generic PM2.5 but a regionally specific carcinogenic mixture, enriched in biomass-burning byproducts, PAHs, and select transition metals. These constituents create oxidative and inflammatory stresses distinct from traffic-dominated urban PM2.5, which is often more metal-rich [7,29,30]. Biomass-derived aerosols are particularly potent in terms of oxidative potential, as demonstrated by recent comparative studies of PM2.5 composition and oxidative stress biomarkers [53]. These data reinforce previous epidemiological reports linking biomass-derived PM2.5 which has been associated with increased mortality and chronic respiratory diseases over the past 20 years [54].

Moreover, Southeast Asia frequently experiences seasonal PM2.5 surges associated with biomass burning and transboundary haze. In Northern Thailand, extreme fire-related pollution episodes have been linked to spikes in hospital admissions and excess mortality [55]. At the global scale, acute pollution episodes of PM2.5 exposure are estimated to have contributed to nearly 694,440 premature deaths between 1990 and 2019 [56]. These real-world surge events closely mirror the acute oxidative stress phenotypes observed in our in vitro models, reinforcing the plausibility that repeated haze exposure may accelerate lung tumor progression in genetically susceptible individuals.

A clinically relevant implication of our findings is the heightened vulnerability of *EGFR*-mutant lung adenocarcinomas, which are disproportionately common in nonsmokers across Asia. In NCI-H1975 cells, PM2.5 exposure enhanced stress-response and survival pathways, suggesting that environmental pollution may exacerbate therapeutic resistance. Prior work has shown that chronic oxidative stress and inflammation remodel the tumor microenvironment in *EGFR*-mutant NSCLC, promoting immune evasion and reduced sensitivity to *EGFR*-targeted therapies [4,57]. These results underscore the importance of region-specific clinical surveillance and support the rationale for combinatorial therapeutic strategies that address both oncogenic drivers and oxidative stress pathways.

From a public health and policy perspective, our findings highlight the limitations of treating PM2.5 as a uniform pollutant. Seasonal haze events in Northern Thailand are recurrent and predictable, yet regulatory frameworks rarely account for chemical composition when setting air-quality standards. Incorporating compositional monitoring into national and regional systems could support more targeted interventions, including biomass-burning controls, sustainable residue management, and haze-alert systems. Such strategies could also inform risk-stratified lung cancer screening, prioritizing vulnerable populations exposed to biomass-derived PM2.5 mixtures.

### 4.5. Limitations and Future Directions

Several limitations should be considered. We used acute, relatively high in vitro PM2.5 concentrations to model surge events, which may not fully capture chronic low-dose exposures. Viability was assessed with a single MTT assay, and oxidative markers were measured in bulk lysates normalized to viable cell number rather than at the single-cell level. Mitochondrial status was inferred from MitoTracker Green and qualitative confocal imaging, which cannot directly quantify membrane potential or mitochondrial-specific ROS. RNA-seq differentially expressed genes were not independently validated by RT-qPCR in this study. However, the transcriptomic patterns were supported by biological replication (n = 3), stringent multiple-testing control, and coherent pathway-level enrichment consistent with the observed oxidative stress phenotypes. Accordingly, we emphasize pathway-level interpretation and present individual genes as candidates for follow-up validation rather than definitive mechanistic proof. work should incorporate chronic exposure models, non-malignant airway or lung epithelial controls, mitochondrial-specific probes (e.g., MitoSOX, TMRM/TMRE, JC-1), and advanced 3D or tumour–immune co-culture systems to more precisely define how biomass-haze PM2.5 perturbs mitochondrial bioenergetics, immune interactions, and therapy responses.

## 5. Conclusions

This study shows that biomass haze-derived PM2.5 from Chiang Mai causes dose- and genotype-dependent cytotoxicity in NSCLC cells, driven by oxidative stress and transcriptional reprogramming. Integrating viability, ROS and MDA measurements, mitochondrial-associated fluorescence, and RNA-seq revealed distinct stress phenotypes: acute but partly reversible oxidative surges in *EGFR*-mutant NCI-H1975 cells, sustained oxidative burden with progressive viability loss in KRAS-mutant A549 cells, and relative resistance in NCI-H460 cells associated with strong induction of xenobiotic metabolism and *TIPARP*-mediated feedback. Across all models, PM2.5 exposure upregulated xenobiotic detoxification genes (*CYP1A1*, *CYP1B1*), stress-adaptive mediators (*GDF15*, *FOSB*, *TIPARP*), and pathways linked to oxidative stress, inflammation, and immune modulation, while repressing tumour-suppressor transcripts (*FAT1*, *LINC00472*). Overall, these mechanistic insights strengthen the biological plausibility that recurrent biomass-burning haze episodes differentially impact lung tumours according to their genetic background and highlight redox-regulated xenobiotic and immune pathways as promising targets for biomarker development and intervention strategies in regions chronically affected by biomass-burning PM2.5.

## Data Availability

The DEG datasets generated and analyzed during this study are provided in Supplementary Materials. Additional raw data are available from the corresponding author upon reasonable request.

## Supplementary Materials

The following supporting information can be downloaded at: https://www.mdpi.com/article/10.3390/toxics14010021/s1, Supplementary Data S1: DEGs_NSCLC_PM.

## Abbreviations

The following abbreviations are used in this manuscript:

PM2.5	Particulate matter with aerodynamic diameter ≤ 2.5 µm
NSCLC	Non-small-cell lung cancer
ROS	Reactive oxygen species
MDA	Malondialdehyde
DEG	Differentially expressed gene
KEGG	Kyoto Encyclopedia of Genes and Genomes
LUAD	Lung adenocarcinoma
LCC	Large-cell carcinoma
AhR	Aryl hydrocarbon receptor
CYP	Cytochrome P450 enzyme family
